# Integrative analysis of gut microbiome and host transcriptomes reveals associations between treatment outcomes and immunotherapy‐induced colitis

**DOI:** 10.1002/1878-0261.13062

**Published:** 2021-07-28

**Authors:** Toshiharu Sakurai, Marco A De Velasco, Kazuko Sakai, Tomoyuki Nagai, Hiroki Nishiyama, Kentaro Hashimoto, Hirotsugu Uemura, Hisato Kawakami, Kazuhiko Nakagawa, Hiroyuki Ogata, Kazuto Nishio, Masatoshi Kudo

**Affiliations:** ^1^ Department of Gastroenterology and Hepatology Kindai University Faculty of Medicine Osaka Japan; ^2^ Department of Genome Biology Kindai University Faculty of Medicine Osaka Japan; ^3^ Institute for Chemical Research Kyoto University Uji Japan; ^4^ Department of Urology Kindai University Faculty of Medicine Osaka Japan; ^5^ Department of Medical Oncology Kindai University Faculty of Medicine Osaka Japan

**Keywords:** *Enterobacteriaceae*, gastrointestinal immune‐related adverse event, immune checkpoint, integrative analysis, microbiome, whole transcriptome

## Abstract

Immune checkpoint inhibitors (ICIs) are widely used to treat various malignancies. Although the gut microbiome is known to influence the efficacy of ICIs on epithelial tumors, the functional interactions between gut taxa and colonic mucosa remain poorly understood. Here we performed transcriptomic profiling and 16S rRNA sequencing to investigate the relationships between mucosal gene expression and microbial composition with ICI responses and gastrointestinal immune‐related adverse events (GI irAEs). In responders, genes related to DNA repair and cell cycle signatures were enriched in responders whereas signatures related to innate immune response, NFAT and IFN‐γ signaling pathways were enriched in nonresponders. Gut microbial composition revealed an association between moderate GI irAE and favorable response to ICI therapy. Favorable therapeutic responses to ICI and GI irAE treatments were associated with taxa classified as *Enterobacteriaceae* and were related to ribonucleoprotein complex biogenesis, cytokine‐mediated signaling pathway, tRNA metabolic process, and ribonucleoprotein complex assembly in the colon. These findings open new perspectives for improving the efficacy and safety of cancer immunotherapy.

## Graphical abstract

In our study, we used an integrative approach to investigate the relationships between mucosal gene expression and microbial composition in patients with cancer that display gastrointestinal immune‐related adverse events (GI‐irAE) after immune checkpoint inhibitor (ICI) therapy. We clustered patients according to their clinical characteristics and identified relevant features associated with response to ICI in inflamed mucosa. Our research indicates that both the host cell transcriptome and the gut microbiome play a key role in immunotherapy‐induced colitis in patients with cancer.

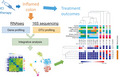

**Abbreviations**
ANOVA: analysis of variance
ChEA3: ChiP‐X enrichment analysis
CR: complete response
CTLA‐4: cytotoxic T‐lymphocyte‐associated protein 4
DEGs: differentially expressed genes
DGE: differential gene expression
FDR: false discovery rate
GI: gastrointestinal
GO: gene ontology
ICI: immune checkpoint inhibition
ICIs: immune checkpoint inhibitors
IFN‐γ: interferon‐gamma
irAEs: immune‐related adverse events
LDA: linear discriminant analysis
LEfSe: linear discriminant analysis effect size
MCODE: molecular complex detection
MDS: multidimensional scaling
NFAT: nuclear factor of activated T cells
NonResp: nonresponders
OTUs: operational taxonomic units
PCA: principal component analysis
PD: progressive disease
PD-1: programmed cell death-1
PD-L1: programmed cell death ligand 1
PR: partial response
RDA: redundancy analysis
Resp: responders
SD: stable disease
TNF: tumor necrosis factor
TRRUST: transcriptional regulatory relationships unraveled by sentence-based text mining
t-SNE: t-distributed stochastic neighbor embedding

## 1 Introduction

Monoclonal antibodies targeting immune checkpoints CTLA‐4, PD‐1 and PD‐L1, referred to as immune checkpoint inhibitors (ICIs), have become a new standard of care in a wide range of cancers [1, 2, 3, 4]. ICIs are distinguished from other targeted therapies and chemotherapies in their mechanism of action. ICI enhances T‐cell antitumor activity while traditional antineoplastic agents exert direct cytotoxic effects [5]. While longer survival is expected in some patients treated with ICIs, these agents have also manifested a new class of immune‐related adverse events (irAE), of which gastrointestinal (GI) irAE are among the most frequent and severe [6]. The onset of GI‐irAE has been attributed to the proliferation and accumulation of cytotoxic CD8 effector cells [7]. However, there is no clear evidence linking the associations between irAE and antitumor effects of ICI.

Commensal microbes are important for well‐being and aid in regulating homeostasis and maintaining healthy immune systems. Gut microbes play particularly important roles and are greatly affected by and contribute to many pathologies [8]. Moreover, longitudinal studies have shown that microbial composition is altered during inflammatory bowel diseases including ulcerative colitis and this dysbiosis may contribute to further disease progression [9, 10]. Recent studies have also described the effects of gut microbes on the efficacy and toxicity of ICIs [11, 12]. In inflamed regions, higher abundances of *Faecalibacterium prausnitzii* or *Akkermansia muciniphila* are associated with an enhanced antitumor response to ICIs [13]. Additionally, recent studies showed that modifying gut microbes could aid in overcoming resistance to ICI [14, 15]. Thus, we hypothesized that associations between irAE grade, ICI efficacy, and microbial composition of colon mucosa exist, and if revealed could further enhance our understanding of ICI.

To achieve our aim, we studied recently diagnosed GI irAE patients who had not yet received drug treatment for colitis and used diagnostic tissue biopsies and fecal samples for whole transcriptome and 16S rDNA sequencing analyses. Here, we survey microbial composition and the transcriptomic landscape of the gut mucosa and perform comprehensive integrative analyses to reveal associations between mucosal microbiota and gene expression profiles with ICI responses.

## 2 Methods

### 2.1 Patients and samples

Patients diagnosed with GI irAEs (*n* = 17) during or after treatment of ICI between March 2017 and March 2019 were included in this study. Clinical, biochemical, endoscopic, and radiological evaluations were performed during follow‐up at the physician’s discretion. Endoscopy was performed, and biopsies were taken from inflamed mucosa for the analysis of gut mucosal microbiota and gene expression profiling. Fecal samples were obtained from 12 GI irAE patients. All samples were immediately dry frozen in nitrogen and further kept at −80 °C before analysis.

GI irAEs were assessed according to the Mayo endoscopic subscore (1: mild, 2: moderate, 3: severe colitis) [16]. The outcome of GI irAE was determined according to response to medical treatments including glucocorticoid and anti‐TNF agents. In the ‘in remission’ group, medical treatments induced clinical remission [16], while in the ‘long‐lasting colitis’ group, patients with GI irAEs were refractory to corticosteroid and TNF blockade and did not achieve clinical remission.

Tumor response was assessed in patients with measurable lesions according to the guidelines of the Response Evaluation Criteria in Solid Tumors version 1.1. Responders were defined as patients who achieved a best overall response of complete response (CR), partial response (PR), or stable disease (SD), while nonresponders were defined as those patients who showed progressive disease (PD).

The study was performed in accordance with the Declaration of Helsinki, with the approval of the ethical committee of Kindai University Faculty of Medicine (#28‐224). All patients provided informed consent prior to enrollment.

### 2.2 Transcriptome analysis

DNA and RNA were extracted simultaneously from the same biopsy samples using the AllPrep DNA/RNA Mini Kit (Qiagen, Valencia, CA, USA). Gene expression was evaluated in inflamed mucosa (*n* = 14) of GI irAE patients using the AmpliSeq Transcriptome Human Gene Expression Kit (Thermo Fisher Scientific,Foster City, CA, USA). Pooled libraries were subjected to the Ion Chef System (Thermo Fisher Scientific) for template preparation. Libraries were then loaded onto an Ion 550 chip and sequenced with the Ion S5 sequencing system. The ion torrent suite version 5.10 software (Thermo Fisher Scientific) was used to map read. Raw read‐count data files were converted to RPKM (reads per kilobase per million reads) for read‐count normalization. Differential gene expression (DGE) analysis was performed by Transcriptome Analysis Console (TAC) software (Thermo Fisher Scientific) with fold change differences > 2.0 or < −2.0. Statistical tests were performed using ANOVA with a *P* value < 0.05 as the significance cutoff, unless otherwise stated. For prediction modeling, gene expression data were filtered and preprocessed as previously described [17]. Summary workflow for transcriptome analysis is shown in Fig. S1.

### 2.3 Microbiome analysis

DNA derived from mucosal samples and feces were processed for 16S rRNA gene amplicon sequencing using the V2‐V4 and V6‐V9 16S rRNA region for single‐end sequencing on the Thermo Fisher Scientific Ion S5 platform (Thermo Fisher Scientific) following the manufacturer’s instructions. Briefly, library preparation for the V2, V3, V4, V6, V7, V8, and V9 16S rRNA region was amplified, followed by end repair and barcoded‐adaptors ligation using the Ion Plus Fragment Library Kit (Thermo Fisher Scientific). The pooled library was then sequenced as single‐end 400‐bp reads using the Ion S5 sequencing kit (Thermo Fisher Scientific). The generated FASTQ files were analyzed using the clc genomics workbench version 12.0 (Qiagen) with the Microbial Genomics Module (Qiagen). Sequence reads were clustered into operational taxonomic units (OTUs) with a 99% identify threshold against the Greengenes database, version 13.8. OTUs were analyzed using calypso (version 8.84) [18]. OTU abundance was normalized with cumulative‐sum scaling (CSS) and log2 transformation. Samples with a total read count < 1000 were filtered yielding 2469 OTUs for subsequent analyses. Hierarchical, correlation, network, similarity, and biomarker analyses were carried out with Calypso Hierarchical radial trees were drawn using an ensemble method based on multiple similarity measures that combined Bray–Curtis dissimilarities with Pearson’s correlation and Spearman’s rho. *P* values obtained for the multiple similarity/dissimilarity measures were combined using the Simes method and corrected for multiple testing by the FDR. Taxa similarity were determined by Redundancy analysis (RDA) using the Bray–Curtis distance metric and significance was determined using the permutation test for constrained redundancy analysis. Taxa associated with response to immune checkpoint inhibition were identified using the linear discriminant analysis (LDA) effect size method (LEfSe) implemented in Calypso using default settings (Kruskal–Wallis test α = 0.05, threshold on the logarithmic LDA score for discriminative features = 2.0). Quantitative heat tree plots were generated in MicrobiomeAnalyst with the r package ‘*metacoder*’ using median abundance between groups at the species level and statistically significant taxa (*P* < 0.05) were identified using the Wilcoxon Rank Sum test [19]. Summary workflow for microbiome analysis is shown in Fig. S1.

### 2.4 Machine learning analysis

Machine learning was carried using Orange an open‐source data mining suite [20]. Data filtering was performed using the built‐in filter widget to remove low count genes with a ~ 30% threshold and after median normalization was applied, the top 5000 most variable genes, based on dispersion, were selected. For the selection of classification features, differentially expressed genes (DEGs) were selected using a two‐tailed *t*‐test and correction for false positive was performed by resampling using the permutation test with α = 0.05 and 50 permutations. The rank widget in Orange was used to select the top 24 ranked genes correlated to ICI response based on an internal chi‐square scoring metric. For the integrative analyses, 5000 genes and 2469 OTUs from the previous analyses were pooled a single data set and the top 2000 features were selected based on ANOVA. We deemed 2000 features were an optimal number to provide classification accuracy while generating appropriately sized clusters of genes and OTUs that could be used to extract biological significance. To obtain these clusters, the selected features were subjected to Louvain Clustering in Orange as an unsupervised method to identify and extract related communities from a large and complex network. The cluster index score was used to as a data attribute and metaclusters were aggregated based on their correlation (Pearson) and Euclidean distance. Distributed Stochastic Neighbor Embedding (t‐SNE, perplexity = 90, PCA components = 8, and using normalized data by subtracting the column mean and dividing by the standard deviation), FreeViz (vector‐based projection), Multidimensional Scaling (MDS, using PCA (Torgerson) initialization), and Isometric maps (Isomap, set to neighbors = 2) were generated in Orange and used for data visualization. Hierarchical clustering and correlation distance maps for the integrative analyses were generated with Orange software and Morpheus (https://software.broadinstitute.org/morpheus).

### 2.5 Gene ontology and functional analysis

Functional analysis of canonical pathways of differentially expressed genes was performed with Metascape (http://metascape.org, [21]). For gene ontology (GO) enrichment analysis, we first identified all statistically enriched terms, accumulative hypergeometric *P* values, and enrichment factors were calculated and used for filtering. Remaining significant terms were then hierarchically clustered into a tree based on Kappa‐statistical similarities among their gene memberships. We then selected a subset of representative terms from this cluster and convert them into a network layout. Terms with a similarity score > 0.3 are linked by an edge (the thickness of the edge represents the similarity score). The network is visualized with cytoscape (version 3.1.2) (https://cytoscape.org/). Then, 0.3 kappa score was applied as the threshold to cast the tree into term clusters. The Molecular Complex Detection (MCODE) algorithm was then used on the relevant network to identify neighborhoods of densely connected proteins. GO enrichment analysis was applied to each MCODE network to assign biological relevance to the network component. Gene‐transcription factors interaction analysis was performed on gene list enrichments to identify upstream transcription factors in the Transcriptional Regulatory Relationships Unraveled by Sentence‐based Text‐mining database (TRRUST).

## 3 Results

### 3.1 Patient characteristics

In total, 17 patients who developed diarrhea and endoscopic findings of GI irAE were evaluated (Table 1). After a diagnosis of ICI‐induced colitis, cancer immunotherapy was stopped in all patients. In 15 of the 17 patients, medical treatments, including glucocorticoid and anti‐TNF agents, induced clinical remission (in remission group). In contrast, three patients were refractory to corticosteroid and TNF blockade, of whom Patient #14 had perforation, Patient #3 underwent ileostomy due to resistance to glucocorticoid, anti‐TNF agents, cyclosporin treatment, and cytoapheresis, and Patient #8 developed long‐lasting colitis (> 1 year) with steroid dependency: the inability of a patient to taper and discontinue corticosteroid without flaring (long‐lasting colitis group). In Patient #2, the outcome of ICI‐induced colitis could not be evaluated due to rapidly progressive cancer.

**Table 1:** Patient characteristics.

	Age	Sex	Primary cancer	ICI	Time to onset (month)	Colitis activity	Treatment for colitis	Outcomes of colitis	Restart of ICI	Time to relapse of colitis	Response of ICI	Other irAE
#1	64	f	Lung	Pem	7	Mild	PSL	In remission	No	N.A.	PD	Pituitary, Liver
#2	61	f	Ovary	Nivo	4	Moderate	PSL	N.E.	Yes	1 month	PD	Skin
#3	63	m	Lung	Pem	3	Severe	PSL, IFX, CyA	Ileostomy	No	N.A.	SD	None
#4	43	m	Kidney	Nivo	3	Moderate	PSL	In remission	Yes	5 months	SD	None
#6	64	m	Lung	Pem	2	Mild	PSL	In remission	No	N.A.	PD	None
#7	79	m	Lung	Pem	3	Severe	PSL	In remission	No	N.A.	SD	None
#8	70	m	Lung	Nivo	3	Mild	PSL	Refractory	Yes	5 months	SD	None
#9	71	m	Lung	Pem	6	Moderate	PSL	In remission	No	N.A.	PR	Lung
#10	62	f	Lung	Nivo	3	Mild	Others	In remission	No	N.A.	PD	None
#11	70	m	Lung	Nivo	3	Moderate	PSL	In remission	No	N.A.	PR	Lung
#12	45	f	Stomach	Nivo	1	Mild	Others	In remission	No	N.A.	PD	None
#13	52	m	Unknown	Nivo	4	Mild	PSL	In remission	No	N.A.	PR	Brain
#14	72	m	Stomach	Nivo	10	Moderate	PSL, IFX	Perforation	No	N.A.	SD	None
#15	70	m	Lung	Nivo	3	Mild	PSL	In remission	No	N.A.	PR	None
#16	70	m	Kidney	Nivo+Ip	1	Moderate	5‐ASA	In remission	No	N.A.	SD	None
#17	69	m	Lung	PDL1	6	Mild	PSL	In remission	No	N.A.	PD	None
#18	74	m	Kidney	Pem	6	Moderate	Others	In remission	Yes	2 months	SD	None

5‐ASA, 5‐amin‐2‐hydroxybenzoic acid; CyA, cyclosporine; f, female; IFX, infliximab; Ip, ipilimumab; m, male; mo, month; mo, month; N.A., not applied; Nivo, nivolumab; PD, progressive disease; PDL1, anti‐PDL1 antibody; Pem, pembrolizumab; Pituitary, pituitary gland; PR, partial response; PSL, prednisolone; SD, stable disease.

Response to ICI therapy was evaluated in 17 patients. Best responses of PR, SD, and PD were observed in 4, 7, and 6, patients, respectively. The feasibility of resuming ICI in patients who discontinue treatment due to irAEs has been debated [22]. In our study, cancer immunotherapy was restarted after the induction of remission in 4 patients and colitis recurred in all the patients within 6 months (1–5 months) after the restart of ICI.

### 3.2 Gut transcriptomes associated with favorable response to cancer immunotherapy

**Fig. 1:**
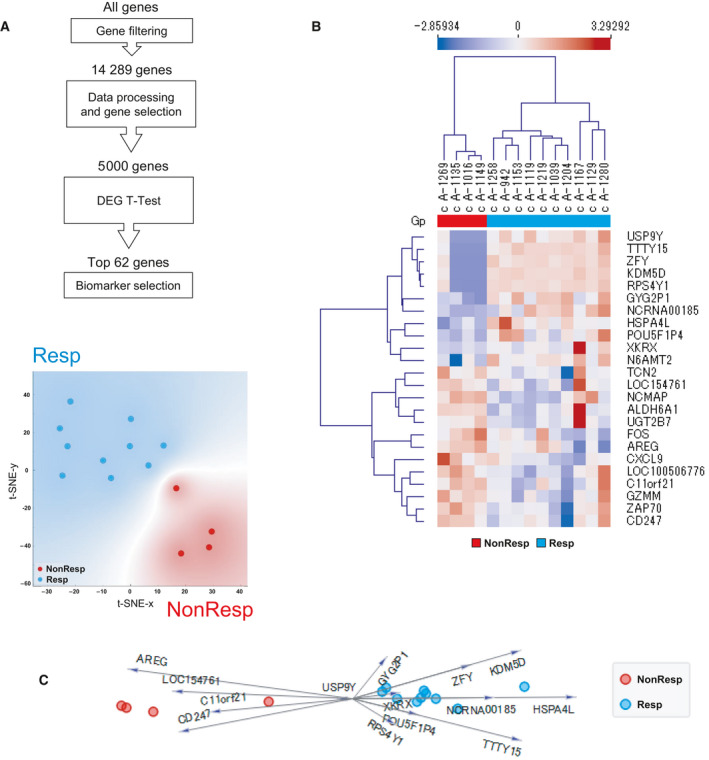
Prediction model that differentiates responders (Resp) and nonresponders (NonResp) to cancer immunotherapy. Gene expression data from next‐generation sequencing of colon from 14 patients were used for prediction modeling. (A) Summary of feature extraction for prediction modeling and t‐SNE visualization with color grouping for patients based on the expression patterns of top 62 differentially expressed genes. (B) Clustering analysis of 24 genes selected for prediction modeling. Unsupervised hierarchical clustering using Euclidean distance and average linkage. (C) Multivariate visualization using FreeViz indicates the 24 most informative genes associated with favorable therapeutic response. DEG, differentially expressed gene; Gp, group; NonResp, nonresponders; Resp, responders.

Our first goal was to develop a model that utilized transcriptomic data to predict favorable responses to ICI. For this purpose, we used machine learning methods to aid us in identifying informative genes that could differentiate responders (Resp) and from nonresponders (NonResp). After initial filtering and feature extraction, 62 genes were selected as candidate classification features (two‐tailed *t‐*test, α = 0.05) and yielded distinct clustering between responder and the nonresponder groups (Fig. 1A). From this set, we used a machine learning algorithm to ranked genes based on their correlation to ICI response and were able to reduce the set of data to the 24 top ranked genes (Fig. 1B). Of note, expression profiling with the 62‐gene signature was not associated with other clinical features such as gender, colitis activity, colitis outcome, primary cancer, and ICI used (Fig. S2). Lastly, we used a multivariate visualization approach to observe the relationships of the genes that are more important for classification (Fig. 1C).

**Fig. 2:**
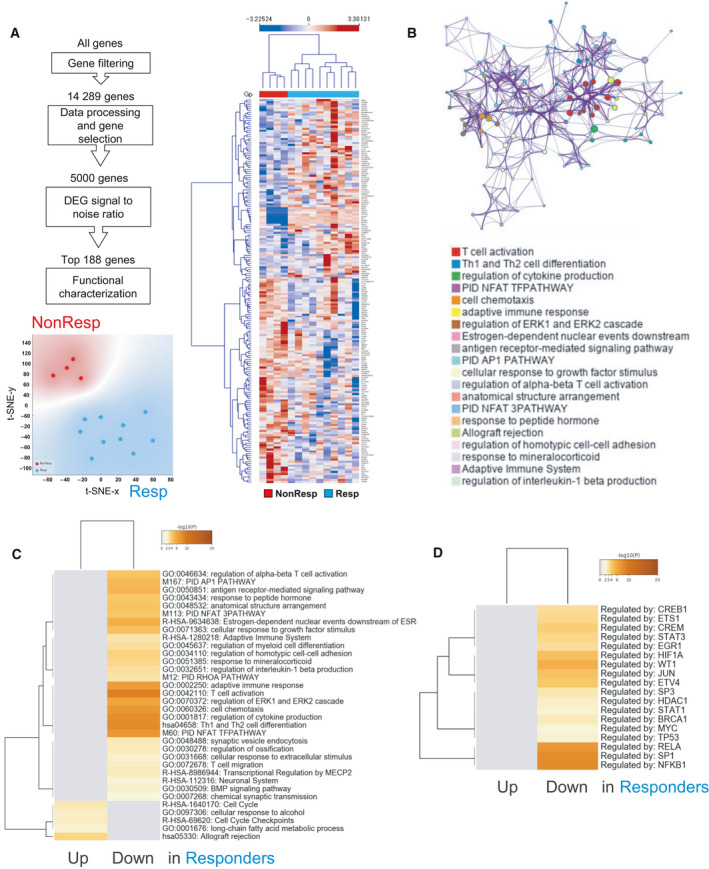
Comparison of gene expression signatures of the rectal mucosa of GI irAE patients between responders and nonresponders to cancer immunotherapy. Gene expression data from next generation sequencing of colon from 14 patients were used for prediction modeling. (A) Summary of feature extraction for prediction modeling, t‐SNE visualization based on the 188 genes, and unsupervised hierarchical clustering using Euclidean distance and average linkage. (B) Networks of enriched terms among the top 188 differentially expressed genes (DEGs) between responders (Resp) and nonresponders (NonResp). Similar nodes are connected by edges, and clusters are coded by color. (C, D) Heat map of enriched terms across input genes (C) and transcription factors (D) in responders. Colors indicate *P* values. DEG, differentially expressed gene; Gp, group; NonResp, nonresponders; Resp, responders.

We next aimed to characterize the functional differences between Resp and NonResp. For this, we selected the top 188 differentially expressed genes and inferred their biological function‐based on gene set enrichments (Fig. 2A). Many of the enriched terms associated with differentially expressed genes were associated with various immune functions including T‐cell activation, Th1 and Th2 cell differentiation, and regulation of cytokine production (Fig. 2B). Statistically significant associations of GO terms and canonical pathways associated with ICI response are shown in Fig. 2C. We also used ChiP‐X enrichment analysis (ChEA3) to identify putative transcription factors regulating the 188 genes. The top 18 transcription factors based on cumulative weighted mean transcription factor ranks of integrated libraries are shown in Fig. 2D.

### 3.3 Survey of gut microbial composition and its association to clinical features in gastrointestinal immune‐related adverse event (GI irAE)

**Fig. 3:**
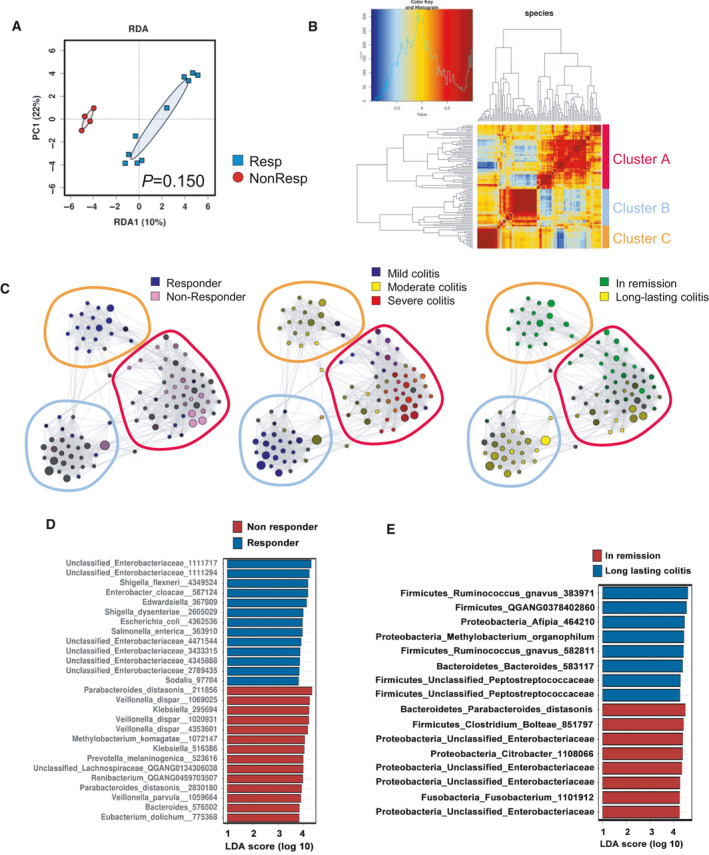
Interrogating the gut microbiome of immunotherapy‐induced colitis. Operational taxonomic unit (OTUs) were clustered from 16S sRNA in colonic tissues from 14 patients. (A) Supervised redundancy analysis (RDA) at the species level. (B) Correlation heat map showing the ensemble similarity distance based the top 100 most abundant species. (C) Correlation networks of taxa according to their association to severity and outcome of colitis and response to immunotherapy. (D) Bar plot of the top 27 OTUs with an absolute LDA ≥ 2 associated with response to cancer immunotherapy identified using the linear discriminant analysis effect size method (LEfSe). (E) Bar plot of the top 16 OTUs with an absolute LDA ≥ 2 associated with the outcome of irAE (in remission *vs*. long‐lasting colitis groups) identified using the linear discriminant analysis effect size method (LEfSe). LDA, linear discriminant analysis; PC1, first principal component, RDA1, redundancy analysis 1.

We next surveyed the gut microbiomes from the corresponding rectal inflamed mucosa samples collected as biopsies. We a used a constrained analysis approach to assess the influence of GI irAE and response to ICI on microbial variation and identified response to ICI therapy as the factor most likely to be associated with bacterial composition (Fig. 3A and Fig. S3A). Further analysis of the top 100 most abundant taxa revealed three distinct clusters of correlated taxa (Fig. 3B). Principal coordinate analysis of taxa using Pearson correlation as a distance metric associated cluster C to moderate colitis and induction of remission (Fig. 3C and Fig. S3B). Interestingly, many of the taxa enriched in cluster C, belong to the family *Enterobacteriaceae,* and included OTUs classified as *Shigella flexneri*, *Citrobacter*, *Klebsiella pneumoniae*, *Enterobacter cloacae*, and other unclassified *Enterobacteriaceae* (Fig. S3B,C). We used linear discriminant analysis effect size (LEfSe) to identify relevant taxa that were associated with or could serve as potential biomarkers for response to ICI therapy (Fig. 3D) and induction of remission of GI irAE (Fig. 3E).

We also examined fecal bacterial composition to determine whether relationships between responses to ICI therapy and GI irAE exist. Overall fecal bacteria showed compositional differences between outcomes of GI irAE and tended to be correlated with the severity of GI irAE and ICI outcome (Fig. S4A). While we did note some clustering of correlated taxa, there was no clear correlation to ICI therapy and GI irAE severity and outcomes (Fig. S4B). Collectively, our findings indicate that mucosal microbial composition is closely related to outcomes from ICI therapy and link moderate colitis to favorable outcomes for ICI and GI irAE treatments.

### 3.4 Integrative analysis of the microbiome and transcriptome in gastrointestinal immune‐related adverse event (GI irAE)

**Fig. 4:**
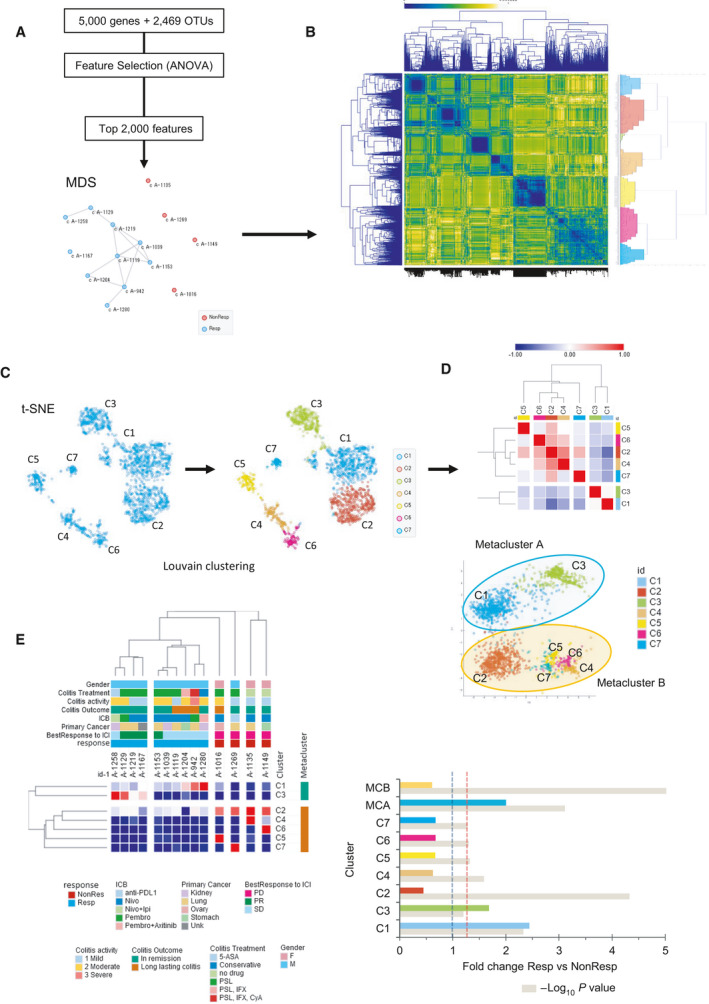
Integrative analysis of the microbiome and transcriptome in gastrointestinal immune‐related adverse events. (A) Overview of feature selection after filtering for variance and multidimensional scaling (MDS) plot showing sample similarity based on selected features. Connected nodes show the greatest degree of similarity. (B) Correlation matrix heat map showing the Euclidean distance between the 2000 selected features. (C) t‐SNE plot showing similarly expressed clusters generated with the Louvain clustering algorithm. (D) Clustered Pearson correlation heat map and isometric map (Isomap) illustrate metaclusters agglomeration. (E) Unsupervised hierarchical clustering using Euclidean distance and average linkage, and bar plot of cluster index scores. Colored bars correspond to cluster and beige bars indicate the ‐log P value, the red dotted line represents the *P* = 0.05 reference, and the gray dotted line represents the reference for NonResp. 5‐ASA, 5‐amin‐2‐hydroxybenzoic acid; CyA, ciclosporin; F, female; ICB, immune checkpoint blockade; ICI, immune checkpoint inhibitor; IFX, infliximab; Ipi, ipilimumab; M, male; Nivo, nivolumab; NonResp, nonresponders; PD, progress disease; Pembro, pembrolizumab; PR, partial response; PSL, prednisolone; Resp, responders; SD, stable disease; t‐SNE, t‐distributed stochastic neighbor embedding; Unk, cancer of unknown origin.

Thus far, our findings indicate that mucosal microbes are closely linked to host immune responses and GI irAEs and may thus be more likely to interact with the host and influence immune response. To further explore this notion, we used machine learning approaches to explore the transcriptomic and metagenomic landscape of clinical features of GI irAEs. Our approach consisted of a supervised approach to extract the top 2000 relevant features (genes and OTUs) from a pool of the top informative genes and OTUs (Fig. 4A and Table S1). Unsupervised clustering of this data set, using Pearson’s distance, revealed seven correlated clusters (Fig. 4B). We used greedy clustering to identify seven clusters of highly interconnected mucosal genes and OTUs that formed two correlated metaclusters (Fig. 4C,D). Unsupervised clustering showed that Metacluster A (containing clusters 1 and 3) was associated with responders to ICI, whereas Metacluster B (consisting of clusters 2 and 4–7) was correlated with nonresponders (Fig. 4E).

**Fig. 5:**
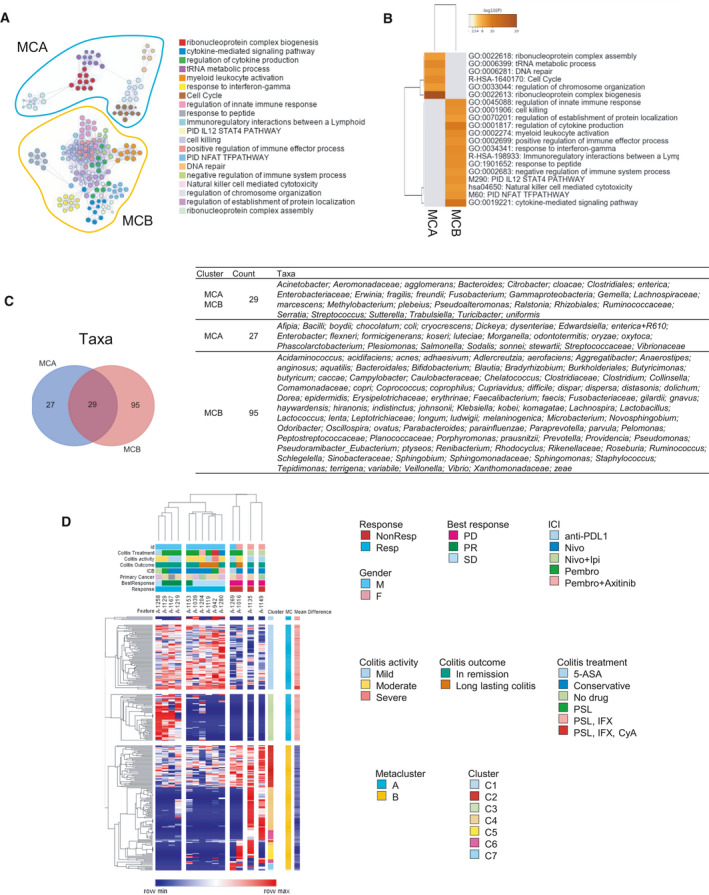
Molecular regulatory mechanisms related to favorable response to immune checkpoint inhibition. (A) Network map of enriched ontology clusters and metacluster association. Similar nodes are connected by edges and clusters are coded by color. (B) Heat map of top enriched terms across input genes for metacluster A (MCA) and metacluster B (MCB), colors indicate *P* values. (C) Venn diagram and table showing species‐level membership of taxa according to metacluster. (D) Unsupervised hierarchical clustering using Euclidean distance and average linkage for individual features and their relationship clinical features. 5‐ASA, 5‐amin‐2‐hydroxybenzoic acid; CyA, ciclosporin; F, female; ICB, immune checkpoint blockade; IFX, infliximab; Ipi, ipilimumab; M, male; Nivo, nivolumab; NonResp, nonresponders; PD, progress disease; Pembro, pembrolizumab; PR, partial response; PSL, prednisolone; Resp, responders; SD, stable disease.

We next performed functional enrichment analysis to gain insights into molecular regulatory mechanisms related to favorable response to ICI. Enriched terms with the best *P*‐values were selected and rendered into networks where terms with a similarity > 0.3 are connected by edges. Enrichments of these network clusters were correlated to the respective metaclusters (Fig. 5A). Among the terms enriched in responders were ribonucleoprotein complex biogenesis, cytokine‐mediated signaling pathway, tRNA metabolic process, and ribonucleoprotein complex assembly (Fig. 5B). We next examined microbial composition in the metaclusters after unifying taxa. Of these, 17.9% (27/151) were unique to Metacluster A and 62.9% (95/151) were unique to Metacluster B (Fig. 5C).

Finally, biomarker selections were performed using the mean difference to identify features that would be most like to be associated with cluster stratification (Table S2). Unsupervised clustering of selected features (genes and OTUs) was performed to explore specific associations with cluster membership and clinical features. Clear clustering was observed between ICI responders and nonresponders (Fig. 5D). Interestingly, high expression of cluster three was associated with patients that achieved PR and had remission of colitis (Fig. 5D). This cluster was predominantly composed of various taxa belonging the *Enterobacteriaceae* family (46 of 51, 90.2%).

## 4 Discussion

Immune checkpoint blockade targeting CTLA‐4 and PD‐1 has become a new standard of care, referred to as cancer immunotherapy. In contrast to the direct cytotoxic action of traditional antineoplastic agents, ICIs enhance antitumor T‐cell activity, which leads to a systemic loss of tolerance, resulting in the occurrence of irAEs. Our study shows that mucosal microbial composition is associated with ICI response and GI irAEs. Findings from our integrative analysis also provide further evidence linking microbial composition to host immune responses and contribute insights into possible molecular events induced by ICI and explore potential treatment strategies to manage GI irAEs.

Given the functional interactions between the gut and systemic immune responses, we hypothesized that gene expression profiling analysis of intestinal mucosal samples obtained from cancer patients could be used to predict the antitumor response to ICIs. We have identified a candidate gene set associated with favorable therapeutic response to ICI. Identification of a panel of genes that is predictive of the therapeutic response would aid in developing new strategies to improve ICI efficacy and clinical outcomes, and however, further validation studies are required. We also hypothesized that an association between GI irAE and ICI efficacy exists. Notably, nine of the 11 responders experienced moderate‐to‐severe colitis, whereas five of six nonresponders experienced mild colitis activity. However, there was no difference in outcomes of colitis treatments between responders and nonresponders. Our observations were consistent with other reports showing positive correlations between ICI responses and the development of GI irAEs [23]. Interestingly, our functional enrichment analysis showed that various genes were associated with immune response processes, including T‐cell migration, and T‐cell activation, were enriched in the intestinal mucosa of patients with unfavorable therapeutic response to ICI. While these results seem to be paradoxical, they indicate that extent of immune responses in the intestinal mucosa is not parallel to that in the tumor microenvironment and could be influenced by other factors.

The gut microenvironment greatly influences the function of the host systemic immune system of the host [24]. Recent studies have described the effects of gut microbiota on the efficacy and toxicity of ICIs [11, 12]. At inflamed regions, high proportions of *Faecalibacterium prausnitzii* or *Akkermansia muciniphila* are associated with an enhanced antitumor response to ICIs [13]. Given that these bacteria augment intestinal inflammation, the previous study suggests that enhanced immune reactions in the gut are associated with increased efficacy to ICI. However, it is not clear as to whether dysbiosis precedes or follows intestinal inflammation. Bacterial composition at inflamed regions might represent dysbiosis resulting from inflammation. Notably, our metagenomic profiling revealed an association between moderate colitis and favorable outcomes to ICI and colitis therapy in mucosal but not fecal samples. Fecal microbial composition tended to be more closely correlated with colitis rather than ICI response. In colonic mucosal samples, an unexpected link was found between *Enterobacteriaceae*, ICI response and remission of colitis. *Enterobacteriaceae* represent a large family of *Proteobacteria* and is composed of Gram‐negative bacteria that includes beneficial commensal and pathogenic organisms. While *Proteobacteria* have been shown to be increased in Crohn’s disease patients, these are not increased in patients with ulcerative colitis [25]. Animal studies have shown that *Enterobacteriaceae* bloom in experimental models of inflammatory bowel disease [25, 26]. Therefore, it may be plausible that increased levels of *Enterobacteriaceae* could be a consequence of GI irAE. However, the fact that these are not abundant in nonresponders indicates a broader interaction between certain taxa in the host gut microbiome and antitumor immunity.

The immune system is modulated by the dynamic interactions occurring between the intestinal microbiome and the host. The importance of interactions between them in the pathogenesis of cancer has become increasingly clear [24]. To better understand the role of the interactions in GI irAE, we used a comprehensive integrative approach to analyze 16S rRNA gene amplicon sequencing with whole transcriptome analysis. By doing so, we have identified modules and networks of similarly expressed genes and intestinal microbes. Based on the integrative analysis of informative genes and OTUs, 7 distinct clusters and 2 metaclusters were identified. Favorable response to cancer immunotherapy was found to be associated with the increase in cell cycle, DNA repair, and regulation of chromosome organization and the decrease in innate immune response, cytokine production, myeloid leukocyte activation, interleukin‐12 (IL‐12)/signal transducer and activator of transcription 4 (STAT4) pathway, and NFAT pathway in rectal mucosa. These results suggest that a regenerative process in response to immune reactions, rather than immune activity itself, might be reflected in GI irAE of ICI responders. Consistent with our previous result, *Enterobacteriaceae* were prominent in responders. While this study provides link between certain taxa in the *Enterobacteriaceae* family and favorable responses to ICI, we cannot establish a causal relationship and will require further study. We also do not know to what degree the associations from our mucosa analyses are exclusive to active colitis.

## 5 Conclusion

This research highlights that both transcriptome and microbiome are key factors in shaping the cancer immunotherapy‐induced colitis, which further enhances our understanding of the host–microbiome interactome involved in irAE pathogenesis. Notably, data from this study shows are that the severity of colitis was associated with a greater objective response in the irAE group suggesting that the moderate‐to‐severe GI toxicities are likely to be associated with ICI responses. Our integrative approach could be used to build a model to predict therapeutic response to cancer immunotherapy and our findings may be used develop novel diagnostic and therapeutic modalities that could enhance cancer immunotherapy and the management of GI irAEs.

## Conflict of interest

The authors declare no conflict of interest.

## Author contributions

TS, MADV, and K Nishio designed the study and wrote the manuscript. TS, MADV, KS, TN, KH, HN, and HO performed data analysis and revised the manuscript. TS, KS, and MADV performed machine learning. TS, MADV, KS, TN, HN, KH, HU, HK, K Nakagawa, HO, K Nishio, and MK contributed data analysis and interpretation of data. K Nishio and TS were involved in project inception and supervision.

### Peer Review

The peer review history for this article is available at https://publons.com/publon/10.1002/1878‐0261.13062.

## Supporting information


**Fig. S1**. Summary workflow for transcriptome and microbiome analysis.

**Fig. S2**. Clustering analysis of 188 genes selected for prediction modeling. Heatmaps show unsupervised hierarchical clustering using Euclidean distance and average linkage for gender, colitis activity, colitis outcome primary cancer, and immune checkpoint inhibitor (ICI).

**Fig. S3**. The gut microbiome of immunotherapy‐induced colitis. (A) Species level redundancy analysis (RDA) according to the severity of colitis. (B) Correlation networks from Figure 3C showing representative species in nodes according to cluster membership. (C) Heat tree analysis showing the pairwise comparison of taxa in non‐responder and responder, and long‐lasting colitis and in remission. Labels represent statistically significant taxa (Wilcoxon *P* value < 0.05) at the species level.

**Fig. S4**. Overview of the fecal microbiome of immunotherapy‐induced colitis. (A) Supervised redundancy analysis (RDA) at the species level. (B) Correlation networks showing associations between the top 100 taxa and clinical features. Resp; responders, NonResp; non‐responders, IR; in remission, LLC; long‐lasting colitis.

**Table S1**. Data matrix of the top 2,000 differentially expressed features between responders and non‐responders.

**Table S2**. Data matrix of candidate biomarker features associated with cluster stratification.

## Data Availability

Raw 16S rRNA gene amplicon sequences will be deposited to DNA Data Bank of Japan/Sequence Read Archive (DDBJ/DRA) under the accession number DRA012351. Additional data are supplied as supplementary material.
