# Supplementary figures and images for: Integrative analysis of gut microbiome and host transcriptomes reveals associations between treatment outcomes and immunotherapy‐induced colitis
Source: Mol Oncol. 2021 Jul 28;16(7):1493–507. doi: 10.1002/1878-0261.13062 (PMC8978521; doi:10.1002/1878-0261.13062)

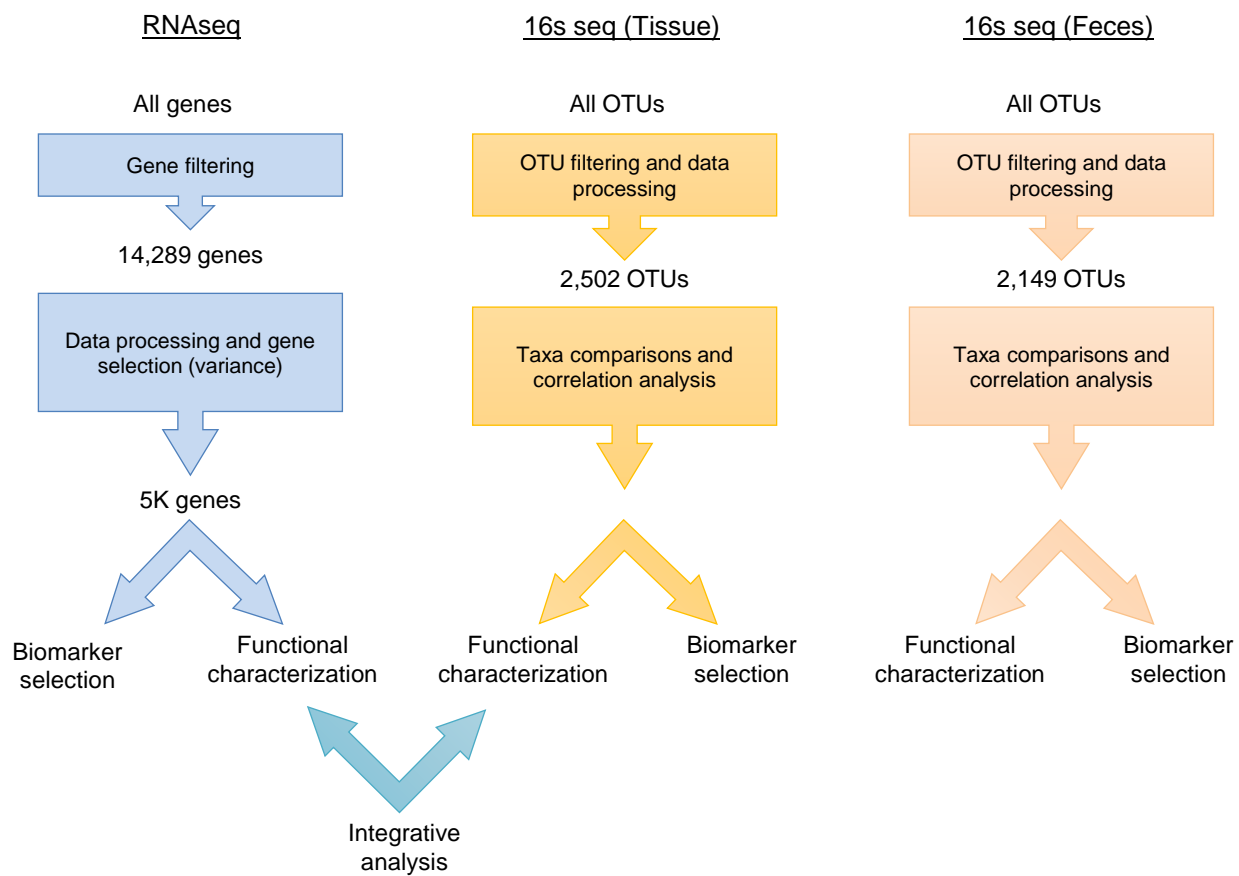

Supplement: Supplementary file 1 — Fig. S1. Summary workflow for transcriptome and microbiome analysis. [file MOL2-16-1493-s003.pdf]

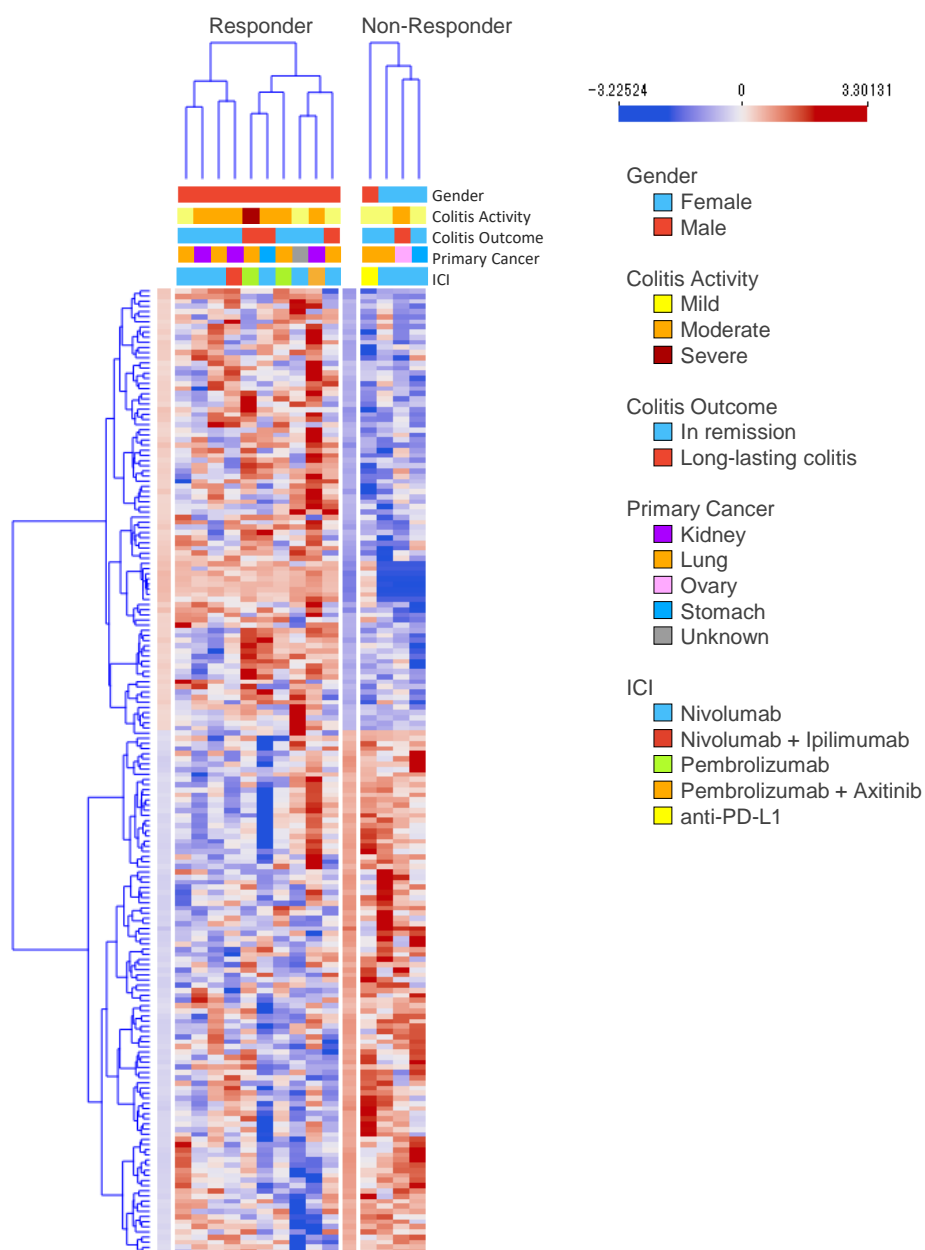

Supplement: Supplementary file 2 — Fig. S2. Clustering analysis of 188 genes selected for prediction modeling. Heatmaps show unsupervised hierarchical clustering using Euclidean distance and average linkage for gender, colitis activity, colitis outcome primary cancer, and immune checkpoint inhibitor (ICI). [file MOL2-16-1493-s001.pdf]

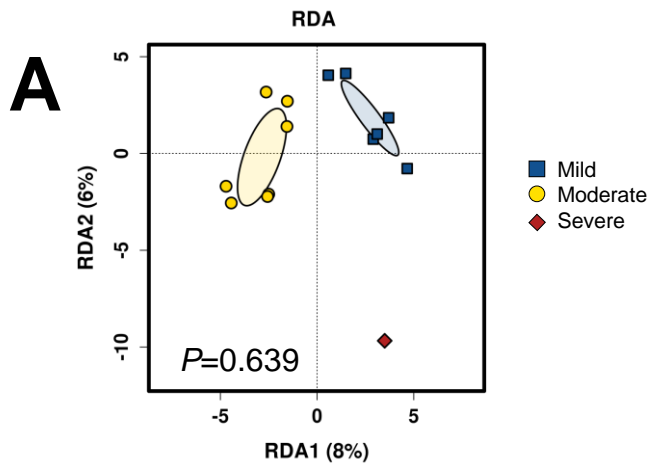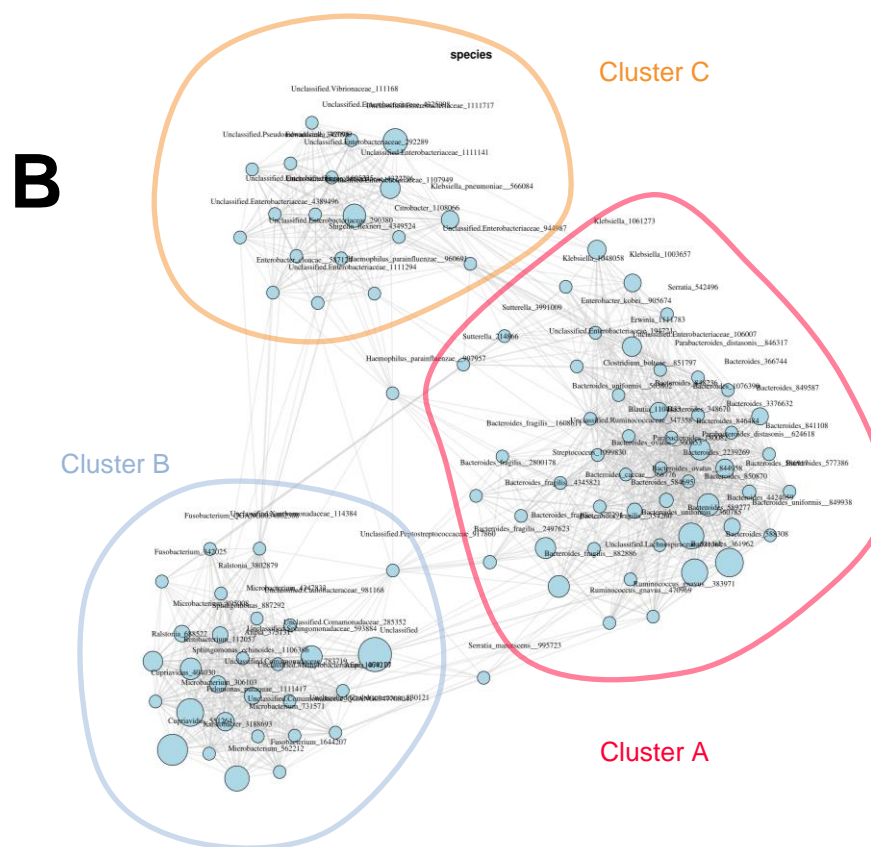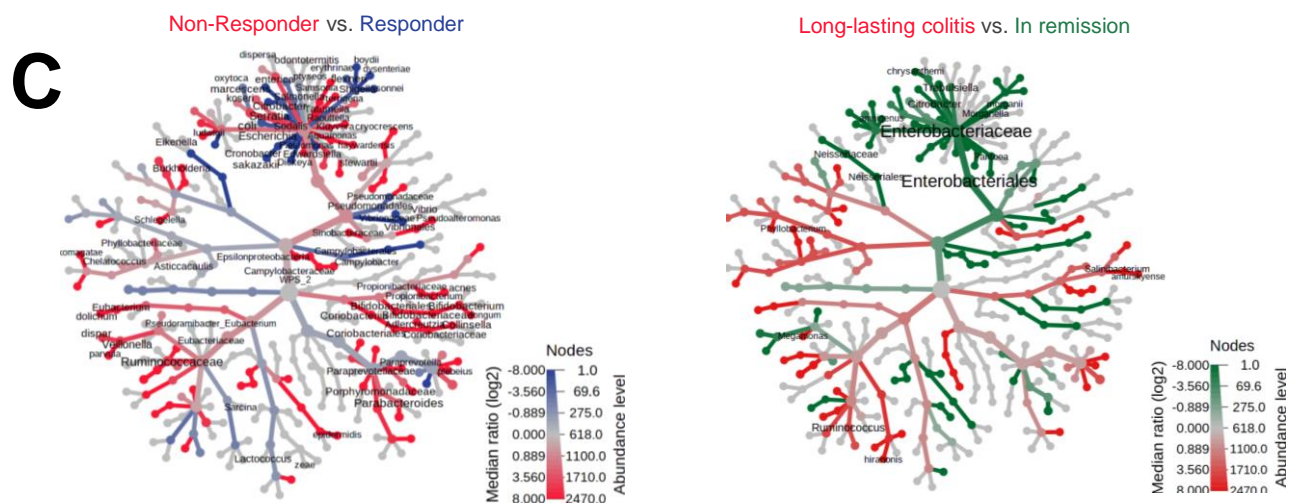

Supplement: Supplementary file 3 — Fig. S3. The gut microbiome of immunotherapy‐induced colitis. (A) Species level redundancy analysis (RDA) according to the severity of colitis. (B) Correlation networks from Figure 3C showing representative species in nodes according to cluster membership. (C) Heat tree analysis showing the pairwise comparison of taxa in non‐responder and responder, and long‐lasting colitis and in remission. Labels represent statistically significant taxa (Wilcoxon P value < 0.05) at the species level. [file MOL2-16-1493-s002.pdf]

# A

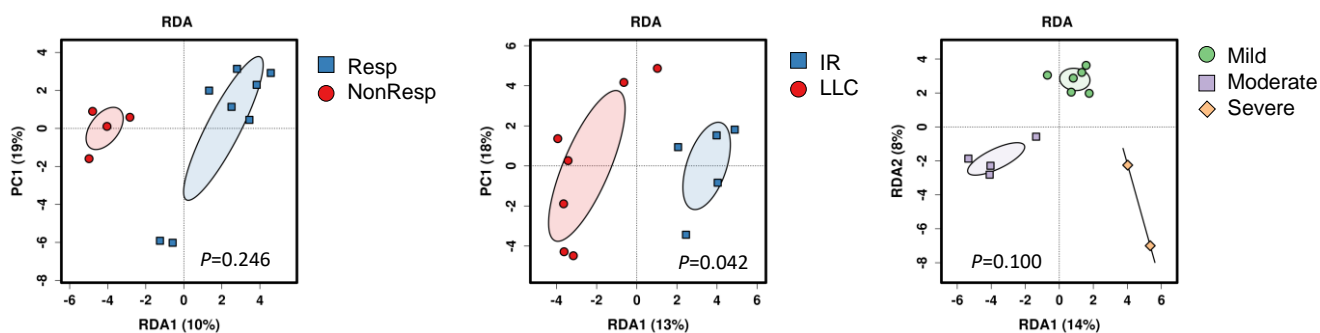

# B

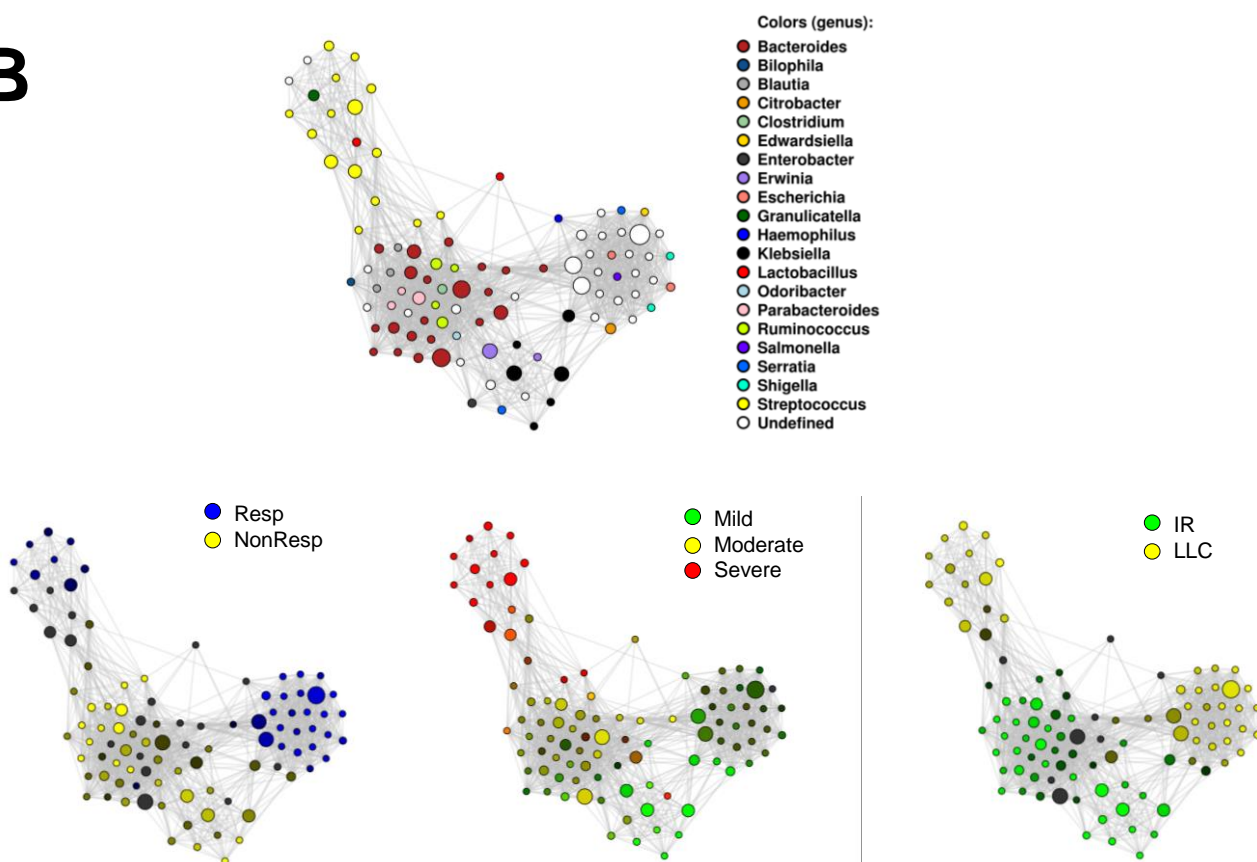

Supplement: Supplementary file 4 — Fig. S4. Overview of the fecal microbiome of immunotherapy‐induced colitis. (A) Supervised redundancy analysis (RDA) at the species level. (B) Correlation networks showing associations between the top 100 taxa and clinical features. Resp; responders, NonResp; non‐responders, IR; in remission, LLC; long‐lasting colitis. [file MOL2-16-1493-s005.pdf]
